# Drug-induced amino acid deprivation as strategy for cancer therapy

**DOI:** 10.1186/s13045-017-0509-9

**Published:** 2017-07-27

**Authors:** Marcus Kwong Lam Fung, Godfrey Chi-Fung Chan

**Affiliations:** Department of Paediatrics and Adolescent Medicine, LKS Faculty of Medicine, The University of Hong Kong, Pok Fu Lam, Hong Kong

**Keywords:** Amino acid starvation, Cancer treatment, Glutamine, Asparagine, Arginine

## Abstract

Cancer is caused by uncontrollable growth of neoplastic cells, leading to invasion of adjacent and distant tissues resulting in death. Cancer cells have specific nutrient(s) auxotrophy and have a much higher nutrient demand compared to normal tissues. Therefore, different metabolic inhibitors or nutrient-depleting enzymes have been tested for their anti-cancer activities. We review recent available laboratory and clinical data on using various specific amino acid metabolic pathways inhibitors in treating cancers. Our focus is on glutamine, asparagine, and arginine starvation. These three amino acids are chosen due to their better scientific evidence compared to other related approaches in cancer treatment. Amino acid-specific depleting enzymes have been adopted in different standard chemotherapy protocols. Glutamine starvation by glutaminase inhibitior, transporter inhibitor, or glutamine depletion has shown to have significant anti-cancer effect in pre-clinical studies. Currently, glutaminase inhibitor is under clinical trial for testing anti-cancer efficacy. Clinical data suggests that asparagine depletion is effective in treating hematologic malignancies even as a single agent. On the other hand, arginine depletion has lower toxicity profile and can effectively reduce the level of pro-cancer biochemicals in patients as shown by ours and others’ data. This supports the clinical use of arginine depletion as anti-cancer therapy but its exact efficacy in various cancers requires further investigation. However, clinical application of these enzymes is usually hindered by common problems including allergy to these foreign proteins, off-target cytotoxicity, short half-life and rapidly emerging chemoresistance. There have been efforts to overcome these problems by modifying the drugs in different ways to circumvent these hindrance such as (1) isolate human native enzymes to reduce allergy, (2) isolate enzyme isoforms with higher specificities and efficiencies, (3) pegylate the enzymes to reduce allergy and prolong the half-lives, and (4) design drug combinations protocols to enhance the efficacy of chemotherapy by drug synergy and minimizing resistance. These improvements can potentially lead to the development of more effective anti-cancer treatment with less adverse effects and higher therapeutic efficacy.

## Background

Uncontrollable cellular proliferation, invasion and metastasis are the characteristics of cancer cells. Due to the absence of cellular contact inhibition, cancer cells can form a huge mass (as in solid tumors) and also migrate to other parts of the body through either blood or lymphatic circulation [1]. In hematologic malignancies, cancer cells will eventually outnumber the normal blood cells by compromising the marrow microenvironment, interfering with the nutrient transport and immune functions of blood and lymph [2]. The malignant tissues in solid tumors invade and damage the surrounding tissues and spread to distant organs, leading to organ failure and death [1]. Cancer cells often have underlying genetic or epigenetic abnormalities affecting both coding and regulatory regions of the genome [3]. These genetic abnormalities cause changes in protein structures, dynamic and expression levels, which in turn alter the cellular metabolism of the cancer cells [3]. The subsequent changes in cell cycles making cancer cells proliferate in a much higher speed than normal counterparts [1]. With the increased metabolic rate and proliferation, the cancer tissues have a much higher nutrient demand compared to normal tissues [4]. As adaptation to fulfill the increased nutritional demand, cancer cells usually up-regulate the glucose and amino acid transporters on the cell membrane to obtain more nutrients from circulations [5]. Cancer cells may even rewire metabolic pathways, usually by enhancing glycolysis (known as Warburg effect) and glutaminolysis, to sustain higher rate of ATP production or energy supply [1, 4, 6]. Glucose and amino acids, especially glutamine, are highly demanded nutrient in cancer cells. Cancer cells are even considered as glutamine-addicting as absence of glutamine supply may induce apoptosis [7–9]. To minimize damages to normal cells, scientists have developed anti-cancer drugs targeting cells with relatively higher metabolic and cellular division rate by different mechanisms. Anti-metabolite is one of these anti-cancer drugs by interrupting the synthesis of bio-chemicals such as nitrogenous bases, nucleotides, or amino acids [10, 11]. In clinical practice, the use of anti-metabolite is more common in hematologic malignancies, but their use in solid tumors is relatively less often [12]. Interestingly, some cancer cell types and tumor tissues are known to be auxotrophic to specific amino acid(s) [13, 14]. Cancers’ auxotrophy to different amino acids may be good druggable targets as they renders the cancer types vulnerable to specific amino acid starvation treatments [15]. This review focuses on the development of different amino acid depletion treatments in treating cancer.

### Responses of cancer cells to amino acid starvation and the molecular mechanisms involved

When mammalian cells experience amino acid starvation, they would undergo a homeostatic response to amino acid shortage [16]. Briefly, with an unclear detection mechanism, amino acid deficiency may trigger general amino acid control pathway [17]. Such pathway involves shifting the resources and energy to expression of membrane transporters, growth hormones, and metabolic enzymes for amino acid homeostasis [17]. Up-regulation of membrane transporters and metabolic enzymes would enhance the amino acid uptake and synthesis, respectively [17].The cells may also recycle proteins and organelles to re-generate non-essential amino acids by autophagy [18]. By general amino acid control pathway and autophagy, the cells attempt to maintain amino acid homeostasis. The tumor tissues can also overcome amino acid starvation by enhancing angiogenesis to obtain more nutrient supply [19]. But when homeostasis cannot be achieved upon severe amino acid starvation, cancer cells may inhibit protein synthesis, suppress growth or even undergo programmed cell death [20]. The cell death mechanisms of amino acid starvation can be caspase-dependent apoptosis or autophagic cell death [21–23]. Amino acid transporters, specific metabolic enzymes, autophagy-associated proteins and even the amino acid itself can all be potential targets for controlling cancer growth. Tremendous effort has been put on glutamine starvation approach by targeting different parts of the glutamine metabolism, that led to the development of specific glutaminase inhibitor CB-839 in clinical trials of different cancers [24]. Clinically, L-asparaginase has been used for depleting asparagine in acute lymphoblastic leukemia [25]. Arginine deiminase and L-arginase are still under clinical trials for their anti-cancer efficacy for arginine depletion [26, 27]. In the following paragraphs, the development of drugs for glutamine, asparagine, and arginine starvation will be discussed.

## Glutamine metabolism inhibition as an anti-cancer strategy

### The role of glutamine in human body

**Fig. 1:**
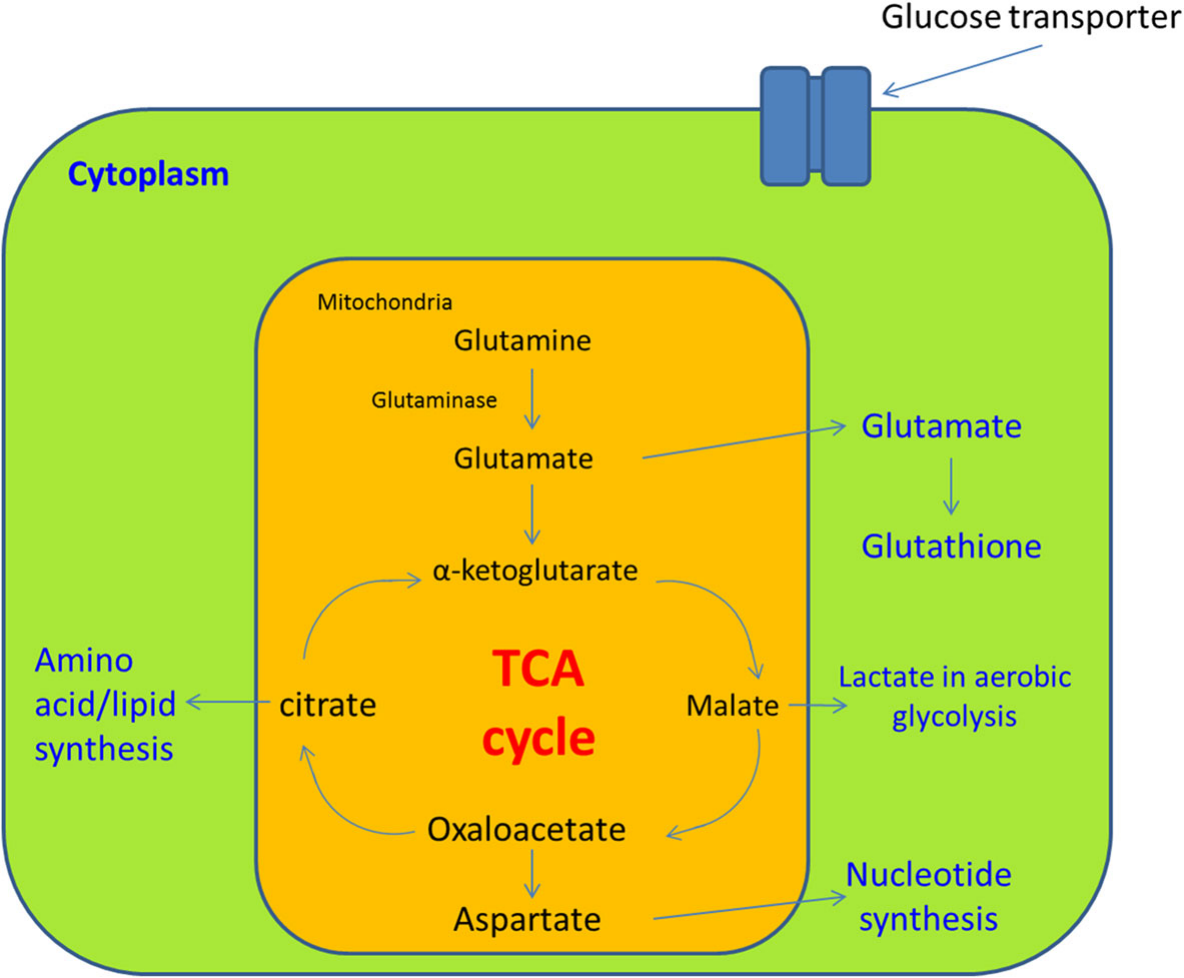
Glutamine metabolism in cancer cells. Glutamine enters the cells through glutamine transporter. After entering the mitochondria, glutamine will be broken down into glutamate by glutaminase. Glutamate can either be transported out to cytoplasm or converted into glutathione. In the mitochondria, glutamate is converted into α-ketoglutarate and enter the tricarboxylic acid cycle. Malate formed in TCA cycle is transported out to the cytoplasm and finally converted into lactate in aerobic glycolysis for energy release. Malate can also be converted into oxaloacetate in mitochondria, which in turn be converted into asparate or citrate. Aspartate is transported out to the cytoplasm for nucleotide synthesis. Citrate formed from malate is transported out to the cytoplasm for amino acid and lipid synthesis

Glutamine is a non-essential amino acid, which means it can be synthesized within human cells. Cancer cells are known to be having enhanced glutaminolysis (conversion of glutamine into glutamate), suggesting glutamine is a very important nutrient for cancer cells [28]. Glutamine has multiple roles in metabolism, from bioenergetics to bio-synthesis of nucleotide, glutathione and other amino acids [29, 30] [Fig. 1]. Glutamine may be converted into α-ketoglutarate for ATP production in oxidative phosphorylation to provide energy for the cells. In cancer cells, glutamine may be equally important as glucose in providing energy depending on cancer type [31]. In impaired glucose metabolism, transamination of glutamine may help to enhance survival as reported in glioblastoma cells in vitro [32]. Glutamine is also precursor of nucleotides and other amino acids for proliferation and glutathione for protection against oxidative stress [33, 34]. Under glutamine depletion, cancer cells may undergo *MYC*-mediated apoptosis [9]. Glutaminase is the key enzyme in breaking down glutamine into glutamate. But glutaminase is considered as a druggable target rather than a candidate for glutamine depletion. High glutaminase expression in tumor tissues may be associated with poor prognosis [35, 36]. The reason may be that high glutaminase expression favors rapid conversion of glutamine to glutamate for use in tricarboxylic acid cycle or bio-synthesis of nucleotides and other amino acids.

### Research targeting on glutamine metabolism inhibition in cancer treatment

Although the importance of glutamine metabolism in cancer has been known for decades, the development of glutamine depletion agents was just started in recent decades. The key concept of glutamine starvation on cancer cells is to stop the cancer cells from obtaining glutamine. The current development of drug targeting on glutamine metabolism of cancer cells focuses on glutamine depletion, glutaminase inhibition, and membrane glutamine transporter inhibition [37–41].

Glutamine depletion was reported effective in suppressing some cancer growth or enhancing cancer-killing activity of immune system [42]. The most well-known glutamine depleting agent is the bacteria-derived L-asparaginase, in which glutamine depletion is the result of off-target [24]. L-asparaginase breaks down both L-asparagine and L-glutamine into L-aspartate and L-glutamate, respectively. L-asparaginase is currently used in treating lymphoblastic lymphoma, acute lymphoblastic leukemia (ALL), and relapsed acute myeloid leukemia (AML) [43, 44]. In the use of L-asparaginase against cancer, glutamine depletion is associated with acute pancreatitis, hepatitis, thrombotic complication, and immune-suppression [44–46]. It is recently suggested that glutaminase activity of L-asparaginase is not always needed for anticancer activity. Asparagine synthetase (ASNS)-negative cancer types, which are defective in asparagine synthesis, are sensitive to L-asparaginase variants without glutaminase activity [47]. However, there are arguments that L-asparaginase together with glutaminase inhibitor may exert a complete inhibition of glutamine metabolism and so enhancing the anti-tumor effect in cancer cells [37, 48].

Other than glutamine depletion, inhibition of glutamine transporter was also studied for anti-cancer drug design, up-regulated glutamine transporter targets SLC1A5/38A2 are recently reported in different cancer types [39, 49]. There are chemicals which may inhibit glutamine transport [50, 51]. Benzylserine was reported to inhibit prostate cancer’s glutamine uptake, metabolism and subsequently tumor growth in vitro, but it requires high dose (at the range of mM) to be therapeutically effective [52]. AOC, chloroalanine and γ-FBP were reported to inhibit SLC1A5-mediated glutamine uptake and cell viability in melanoma cell in vitro, just like benzyl serine, the effective doses were apparently too high for clinical application(at the range of mM) [50].

Advanced development of glutamine starvation approach has been focusing on the specific glutaminase inhibitors, 968, BPTES and CB-839, in which the anti-cancer activity have been studied in vitro or in vivo [40, 41, 53–59]. Both BPTES and 968 were reported to be having low micromolar potency and chemical modification may be need for improvement [60]. In animal studies, BPTES and CB-839 did not show any apparent toxicity [41, 58]. However, CB-839 was reported to be more potent than BPTES in glutaminase inhibition [41]. Recent development of a novel BPTES formulation, the BPTES nanoparticles (BPTES-NPs) were reported to have anti-tumor activity comparable to CB-839 in an orthotopic pancreatic cancer mouse model [61]. BPTES-NPs is produced by encapsulating high dose of BPTES into biodegradable nanoparticles composed of block copolymers of poly(lactic-coglycolic acid) and poly-ethylene glycol [61]. BPTES-NPs treatment on mice did not elevate the plasma level of liver enzymes which CB-839 treatment did, implying a better safety profile [61]. In that report, BPTES-NPs also synergized with metformin to reduce tumor growth by blocking both glucose and glutamine metabolism of cancer cells [61]. This report provides a clue that chemical modification by nanoparticle encapsulation may be a way to enhance the potency of the drugs. Recent clinical trials on glutamine metabolism inhibition mainly focuses on using CB-839 to target glutaminase (NCT02071862, NCT02071888 and NCT02071927 for solid tumors, lymphoid, and myeloid malignancies respectively) [24, 62]. In a phase I trial of CB-839 in relapsed leukemia, patients receiving CB-839 treatment usually showed grade 1 or 2 toxicities like transaminitis, thrombocytopenia, gastrointestinal events, and fatigue and about 1/3 (9/26) patients showed grade 3 or 4 toxicities like hematologic cytopenia [63]. In another phase I trial of CB-839 and paclitaxel in triple negative breast cancer, patients receiving CB-839 and paclitaxel showed different extents of improvement from disease control to partial response with tolerable adverse effects [64]. These clinical trial findings suggest CB-839 may be a safe drug to be used for glutamine metabolism inhibition in cancer treatment.

### Glutaminase inhibitor resistance and ways to enhance the efficacy for cancer treatment

Like any other chemotherapeutics, cancer cells may have resistance to glutaminase inhibitors. There are two glutaminase inhibitor resistance mechanisms reported [58, 65]. One is the over-expression of GLS or GLS gene variant GLS-K325A leading to BPTES and CB-839 resistance in P493 lymphoma cell line and PC3 prostate cancer cell line respectively [58]. The report also showed that genetic inhibition of human GLS mRNA expression in subcutaneous tumor mouse model would also lead to tumor growth suppression [58]. These findings imply a novel therapeutic approach of inhibiting GLS gene expression when the cancer cells have GLS mutant gene or GLS over-expression. Another glutaminase inhibitor resistance mechanism is associated with asparagine auxotrophy. CB-839 resistant breast cancer cell line (but not their parental cells) showed down-regulated glutamine consumption and requires exogenous asparagine in cell culture medium for proliferation, implying glutamine independence in cancer cells may be related to the switch to asparagine pathway in order to reach the cellular demand for survival [65]. The Author then suggested that coupling GLS inhibition to low-asparagine diet may improve the efficacy of GLS inhibitors [65]. Table 1 summarizes the drug development of glutamine depletion approach. In summary, glutamine is a very important substrate in cancer survival, which makes glutamine metabolism a potentially druggable target. Combination of glutamine metabolism inhibition with other drugs may help in improving the therapeutic efficacy. Further investigation in both animal studies and clinical trials of different cancer types may help to determine the exact efficacy and safety profile of glutamine metabolism inhibition in cancer treatment.

**Table 1:** Current development of glutamine metabolism inhibition in treating cancer

Approach	Drug used	Cancer type tested and progress	Reference
Glutamine depletion	No specific glutamine depleting agent available, L-asparaginase acts as both L-glutamine and L-asparagine depleting agent (more detailed discussion in “Asparagine starvation”)	1. Clinical use in treating specific hematologic malignancies, glutamine depletion considered as an off-target effect (Anti-cancer efficacy of L-asparaginase to be discussed in the part of L-asparagine depletion)	[37, 48]
		2. Glutamine depletion by methionine-L-sulfoximine suppressed sarcoma growth in vitro and HCC growth in vivo (subcutaneous (s.c) athymic mouse model)	
Glutamine transporter inhibition	Specific inhibitor not yet available, benzylserine may inhibit one of the glutamine transporter SLC1A5	Benzylserine inhibited prostate cancer in vitro and in vivo (s.c. athymic mouse model)	[52]
Glutaminase inhibition	CB-839 (Glutaminase-1 specific)	1. Anti-proliferative effect on selected breast cancer cells in vitro and in vivo (s.c athymic mouse model), both as single agent or in combination with paclitaxel	[41, 62, 160, 161]
		2. CB-839 synergizes with erlotinib to induce apoptosis in EGFR-mutated non-small cell lung cancer in vitro and reduced tumor growth in vivo (s.c. SCID mouse model)	
		3. CB-839 synergizes with Bcl-2 inhibitor ABT-199 in killing AML blasts in vitro and in vivo (NOD/SCID γleukemic mouse model)	
		4. CB-839 synergizes with carfilzomib in killing proteasome inhibitor resistant myeloma cell lines in vitro	
	BPTES (Glutaminase-1 specific)	1. Growth suppression in glioma cells with IDH-mutation in vitro	[53, 55, 58]
		2. Growth suppression in acute myeloid leukemia cells with IDH-mutation in vitro	
		3. Caused lymphoma cell death in vitro	
		4. Prolonged mice survival in subcutaneous HCC and lymphoma model (s.c athymic mouse models)	
	BPTES nanoparticle	1. Intravenous BPTES-NP injection caused drug concentration in pancreatic cancer cells in vivo (orthotopic athymic mouse model)	[61]
		2. BPTES-NP significantly reduced G2/M/S cycling cells but not hypoxic cells in vivo.	
		3. BPTES-NP combined with metformin could enhance tumor suppression in vivo by simultaneous inhibition of glucose and glutamine metabolism.	
	DON (Target glutaminase-1, may also target glutamine fructose-6-phosphate amidotransferase)	DON: 1. Suppressed growth in colorectal cancer cells in vitro	[36, 162]
		2. Suppressed the growth and metastasis of subcutaneously implanted athymic mouse brain tumor	
	Alkyl benzoquinones, (Glutaminase-2 specific inhibitor)	Reduced proliferation and anchorage-independent colony formation and induce autophagy in liver cancer cells in vitro	[40]
	968 (Glutaminase-1 specific)	1.Inhibited growth of oncogenic fibroblast, breast cancer and lymphoma cell lines in vitro through inhibition of glutaminase	[54, 56, 57, 59, 163]
		2. Inhibited lymphoma growth in vivo (s.c. implanted lymphoma cell line in SCID mice)	
		3. Induced G1 phase cell cycle arrest, cellular stress and apoptosis and sensitized cells to anti-proliferative effect of paclitaxel in human ovarian cancer cell lines in vitro	
		4. Inhibited migration, proliferation and autophagy in non-small cell lung cancer in vitro, 968 combined with CQ further enhanced cell growth	
		5. Reduced the reactive oxygen species elimination capacity to potentiate the cytotoxicity induced by dihydroartmesinin in HCC in vitro	

## Asparagine starvation as an anti-cancer strategy

### The role of asparagine in human body

**Fig. 2:**
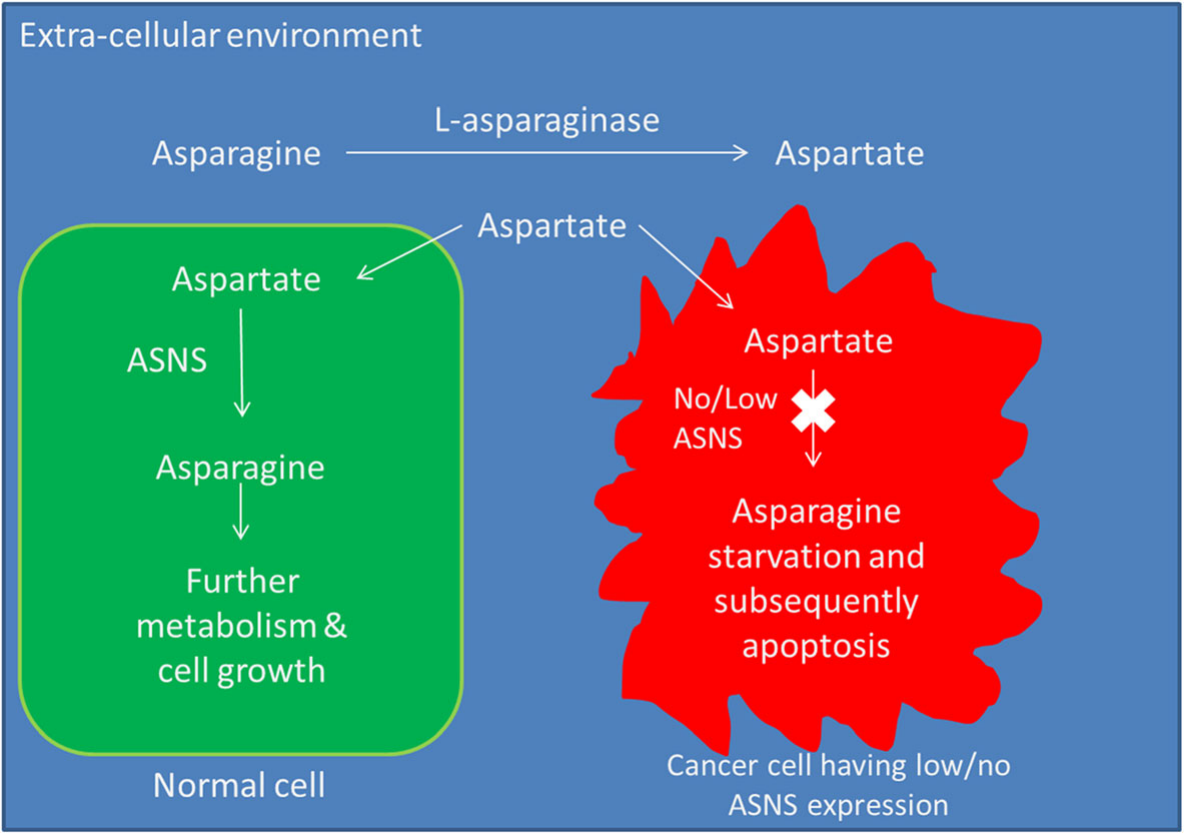
Concept of asparagine starvation in cancer treatment using L-asparaginase. During L-asparaginase treatment, L-asparagine in blood circulation will be broken down into L-aspartate. L-aspartate enters the cells through amino acid transporter. In normal cells, L-aspartate will be converted back into L-asparagine by L-asparagine synthetase (ASNS) for further use. However, some cancer cell types may have no or little ASNS expression and they cannot produce asparagine for further use. They will suffer from asparagine starvation and subsequently undergoing apoptosis

Asparagine is a non-essential amino acid for normal human cells, which means that human cells can synthesize asparagines by themselves. Inside the cells, asparagine is the precursor of aspartate for conversion into malate as a tricarboxylic acid cycle intermediate for respiration or acts as a neuro-transmitter in neuro-endocrine tissues [66]. Asparagine is important in protein synthesis. The amide nitrogen of the asparagine residue may allow N-linked glycosylation, contributing to protein’s structural determination [67, 68]. Asparagine acts as an amino acid exchange factor regulating uptake of amino acids like arginine, histidine, and serine [65]. Asparagine may also coordinate the protein and nucleotide synthesis through the regulation of mTORC1 activity [65]. Cancer cells have increase demand for glutamine, while asparagine can suppress apoptosis induced by glutamine starvation [69]. Asparagine suppresses the endoplasmic reticulum stress and regulates translation-dependent apoptosis during glutamine starvation, suggesting that asparagine may be a suppressor of apoptosis in cancer [69]. In some cancer cells, the expression of ASNS for synthesizing asparagine from aspartate is low or even absent and such cancer cells may require external source of asparagine. L-asparagine depletion by L-asparaginase may induce apoptosis on such cancer cells [70].The defect of cancer cells in asparagine metabolism makes the asparagine pathway potentially druggable. The concept of asparagine starvation as an anti-cancer approach is illustrated in Fig. 2.

### Research progress of L-asparaginase in cancer treatment

The study of asparagine metabolism inhibition for cancer treatment can be traced back to four decades ago. It had been known that leukemia and lymphoma require asparagine for growth in cell culture due to absence of ASNS expression [71, 72]. A study showed that guinea pig serum has anti-tumor activity in lymphoma in vitro and in vivo [73]. Later scientists discovered such anti-tumor activity was due to the high serum level of L-asparaginase [74, 75]. Scientist later discovered and isolated *E. coli* L-asparaginase which had the same anti-tumor activity as guinea pig serum [76]. Such breakthrough led to the successful use of bacteria-derived L-asparaginase in treating acute lymphoblastic leukemia (ALL) [76]. Currently, L-asparaginase is the only asparagine-metabolism targeting agent used clinically. L-asparaginase is a standard induction agent in all conventional protocols for childhood acute lymphoblastic leukemia (ALL) and lymphoblastic lymphoma. Under some circumstances, it is also included in the relapsed protocol for acute myeloid leukemia (AML) [43, 44]. L-asparaginase is a metabolic enzyme with dual asparaginase and glutaminase activity. It breaks down asparagine and glutamine into aspartate and glutamate, respectively. It is believed that L-asparaginase targets cancer cells which have no ASNS expression for synthesizing asparagine from aspartate. The glutaminase activity in L-asparaginase is believed to exert anti-cancer effect on ASNS-expressing cells [47]. There had been pre-clinical studies showing that the anti-tumor activity of L-asparaginase actually covers a wide range of cancer types. In vitro experiments showed that L-asparaginase induced apoptosis in sarcoma and suppressed angiogenic potential as well as inducing autophagy in ovarian cancer [48, 77]. L-asparaginase also synergizes with other drugs to give enhanced anti-cancer effect. Doxorubicin together with L-asparaginase significantly increased the number of cell death in breast cancer in vitro [78]. L-asparaginase in combination with temozolomide suppressed subcutaneous glioma growth in mice [79]. Even though there are reports of anti-tumor activity in L-asparaginase in a wide range of cancers, L-asparaginase has been proven clinically beneficial only in ALL and some lymphomas but not the others. It is because the adverse effects (such as anaphylaxis, thromboembolism, and pancreatitis) of L-asparaginase may outweigh the benefits [80]. In an early study, the use of L-asparaginase as single agent in treating ALL and lymphosarcoma was reported to achieve complete remission in some patients [81]. Combination drug therapy using L-asparaginase, vincristine and prednisone further enhanced the rate of complete remission in ALL treatment [82]. Currently, vincristine and L-asparaginase are included in almost all standard childhood ALL chemotherapy protocols with >90% complete remission rate achieved. L-asparaginase is an essential part for all childhood ALL therapy protocols [25, 83].

### L-asparaginase resistance

Despite high remission rate, relapse is still common in ALL patients. L-asparaginase resistance is one of the important reasons for relapse. There are different resistance mechanisms reported in both pre-clinical and clinical studies. Normal and leukemic lymphoblasts may degrade L-aspraginase and potentiate antigen processing, leading to immune reactions against L-asparaginase [84]. Production of neutralizing anti-asparaginase antibody in response to the foreign protein L-asparaginase may contribute to drug inactivation [85, 86]. Other than immune reactions, it had been a general belief that high ASNS protein expression in some of the leukemia blasts contribute to L-asparaginase resistance [87, 88]. L-asparaginase associated with high ASNS expression may be overcome by the use of ASNS inhibitor as reported in an in vitro study of L-asparaginase resistance leukemia cell line [89]. But such hypothesis is not yet verified in clinical situation. Different in vitro experiments demonstrate other mechanisms proposed for explaining the L-asparaginase resistance. Most of these are related to the intracelullar changes in the cancer cells. Increased activity of glutamine transporter and glutamine synthetase through post-translational modification may enhance the L-asparaginase resistance of cancer cells [90]. The L-asparagine resistance associated with high glutamine synthetase expression may also be overcome by using glutamine synthetase inhibitor and this requires further investigation [91]. Cancer micro-environment may also contribute to L-asparaginase resistance in leukemic blasts. Mesenchymal stromal cell (MSCs) is one of the important components in cancer micro-environment [92]. A transwell co-culture study between bone marrow-derived MSCs and B-lineage ALL blasts shows that bone marrow-derived MSCs may secrete L-asparagine and rescue leukemic blasts from L-asparaginase cytotoxicity [93]. Such finding provides an insight that cancer micro-environment may provide chemo-resistance to leukemic blasts and they may also be druggable targets. We previously reported that MSCs is resistant to most of the chemotherapeutics but sensitive to micro-tubule targeting agents like paclitaxel and vincristine [94]. Later we reported that vincristine pre-treatment on MSCs suppressed the protective effect of MSCs to B-lineage ALL blasts during L-asparaginase treatment [95]. This may explain the synergistic effect of vincristine and L-asparaginase, in which vincristine may suppress the protective effect of MSCs in bone marrow to ALL blasts during L-asparaginase treatment. There is report suggesting that asparagine level is low after induction treatment of ALL, this in fact verified our view that the use of vincristine before L-asparaginase can suppress MSCs from producing asparagine. With all these complex mechanisms involved in L-asparaginase resistance, it further strengthens our belief in using combination of chemotherapy rather than a single drug in treating cancers.

### Side effects of L-asparaginase and possible solutions

Not only drug resistance to L-asparaginase is an issue, therapy related side effect is another problem. Glutamine depletion is a common side effect of L-asparaginase due to its dual asparaginase and glutaminase activity. Glutamine depletion may cause acute pancreatitis, thrombotic complication, and immune-suppression [44–46]. The glutaminase activity of L-asparaginase is not required for anti-cancer activity against ASNS-negative cancer cells, therefore there have been attempt for purifying glutaminase-free L-asparaginase from other sources or engineering L-asparaginase to be glutaminase-free [96, 97]. On the other hand, origin of L-asparaginase used may also contribute to side effects. L-asparaginase currently used in chemotherapy comes from bacterial origin. When L-asparaginase is injected to the human body, the human body may respond by producing antibodies leading to drug hypersensitivity or even anaphylaxis [98]. To tackle these problems, clinicians may select different L-asparaginase formulations depending on situations. Currently there are three formulations of L-asparaginase available for clinical use. They are native *E. coli* L-asparaginase, pegylated E. coli L-asparaginase and native *Erwinia chrysanthemi* L-asparaginase [99]. These three formulations differ from each other in their half-life, immunogenicity, and toxicity. These three formulations are using L-asparaginase from different origin, either from *E. coli* or *Erwinia chrysanthemi*. Pegylated form of *E. coli* L-asparaginase involved the addition of polyethylene glycol group into the native enzyme that can reduce immunogenicity and prolonged half-life (1.24 day for native and 5.73 days for pegylated formulation) [100]. However, the antibodies induced by native *E. coli* L-asparaginase may cross-react with pegylated *E. coli* L-asparaginase [101]. This may contribute to allergic reactions and also drug resistance. To deal with drug resistance due to anti-asparaginase antibody, *Erwinia chrysanthemi* L-asparaginase is an alternative to *E. coli*-origin L-asparaginase. It may induce less complications and toxicities (like coagulation abnormalities, neurotoxicity, and pancreatitis) than L-asparaginase in leukemia patients [102, 103]. However, the half-life (1.24 day for *E. coli* vs 0.65 day for *Erwinia*) of *Erwinia* L-asparaginase may be much shorter than that of *E. coli* L-asparaginase [104]. It was reported that antibodies induced by *E. coli* L-asparaginase would not cross-react with *Erwinia* L-asparaginase [101]. Therefore, it is suggested that *Erwinia* L-asparaginase is preferred for patients with allergy to *E coli* L-asparaginase [105]. Pegylated *Erwinia* arginase is also developed recently for improving the clinical efficacy of *Ewinia* L-asparaginase [106]. But, it is still not yet approved by government authorities for clinical use [106]. After all, native and pegylated *E. coli* L-asparaginase are still the first-line drug while Erwinia L-asparaginase is used when patients show allergic responses to native and pegylated *E. coli* L-asparaginase.

In summary, L-asparagine depletion approach by L-asparaginase treatment may suppress cancer growth in different cancer types under pre-clinical settings. Exploring the chemo-resistance mechanism(s) of cancer cells to L-asparaginase may help in designing or improving multi-drug chemotherapy protocols. After weighing the therapeutic benefits and the adverse drug effects, L-asparaginase seems to be clinically beneficial only in treating selected hematologic malignancies. To reduce the side effects and optimize the benefits on patients using L-asparaginase, it is important to develop versions of L-asparaginase with longer half-life, low-immunogenicity, and low glutaminase activity. Allergy test before treatment and monitoring of blood anti-asparaginase antibodies level during treatment may also help to manage the complications caused by the use of L-asparaginase. Resolving the problem of anaphylaxis may allow the use of L-asparaginase in treating a broader spectrum of cancer types. To resolve the problem of immunogenicity and anaphylaxis-related complications, formulation of human-derived L-asparaginase may be a solution in the future.

## Arginine starvation as an anti-cancer strategy

### The role of arginine in human body

**Fig. 3:**
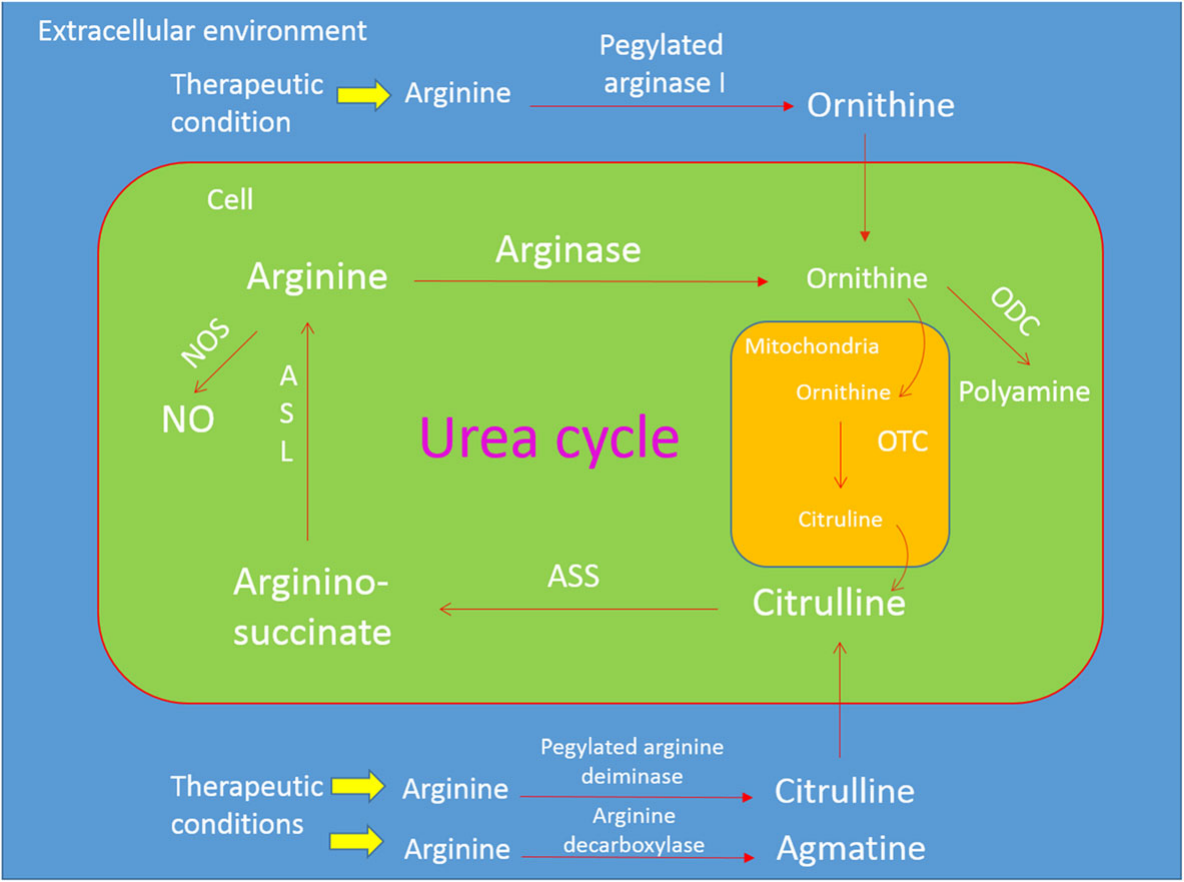
Arginine metabolism in human cells. Arginine may be used to synthesize nitric oxide by nitric oxide synthase ubiquitously. Liver and kidney (to a much lesser extent) are the major sites of urea cycle, mainly for detoxification of ammonia. Arginine is broken down into ornithine by arginase. In liver or kidney cells’ cytosol, arginine is regenerated in the urea cycle. Ubiquitously, ornithine can be converted by ornithine decarboxylase into putrescine for polyamine synthesis. Depending on the types of arginine depleting agents, arginine can be broken down into different intermediates in extra-cellular environment by the corresponding drug mechanisms. Arginine is broken down into agmatine, citrulline or ornithine by arginine decarboxylase, arginine deiminase, and arginase, respectively

Arginine is a versatile semi-essential amino acid. It can be synthesized from the body using glutamine, glutamate and proline in adult but not childhood [107]. Dietary intake is still the major source of arginine as endogenous bio-synthesis may not provide adequate supply [108]. Arginine metabolism is complex and it has diverse roles like protein structure determination, precursors for signaling molecules, urea cycle intermediates, and tri-carboxylic acid cycle intermediates [109–111]. The bio-chemical pathways of arginine metabolism are illustrated in Fig. 3.

### The role of arginine derivatives in cancer

The role of arginine in cancer has been explored for quite a long time. Way back in the 50s, arginine had been shown to have a bipolar effect in either growth stimulation (for large tumors) or inhibition (for small tumors) in different rat sarcoma models [112]. This suggests arginine has a diverse role in cancer development. Arginine is the precursor of cancer-associated factors like nitric oxide (NO) and polyamine families, which are also ubiquitously produced in human tissues [113–115]. Arginine is the only substrate of nitric oxide synthases (NOS), which generate NO [110]. The role of NO in cancer is conflicting and is possibly dependent on concentration, effector cell types, and duration of exposure [14]. In general, low concentration (in nano-molars) of NO may be tumoriogenic [116]. It may promote carcinogenesis, enhance cancer cell growth, and also enhances angiogenesis to favor tumor progression [14]. However, it is suggested that high concentration (in micro-molars) of NO may induce apoptosis in cancer cells by damaging DNA, although there is no corresponding clinical observation [117, 118]. High polyamine bio-synthesis activity had been reported in hepatoma and sarcoma in vitro [119]. Elevation of urine and erythrocyte polyamine was reported in patients with different cancers like stomach, lung, colon, liver cancer, and blood cancers [120, 121]. Elevated tissue ODC activity and polyamine level are associated with breast, colon, skin, and prostate cancer [122–126]. There had been different reports about polyamine enhancing the proliferation of cancers like breast, colon, lung, prostate, and skin cancers [125]. Arginine deprivation may affect actin cytoskeleton organization due to β-actin arginylation impairment in a glioblastoma in vitro model [111]. These findings suggest an important role of arginine and its metabolites in cancer progression. Cells lacking OTC and ASS-1 expression may be auxotrophic to arginine. It was reported that some cancer types are lacking OTC and/or ASS1 expression, so they may be auxotrophic to arginine (http://www.proteinatlas.org). Responses of cancer types sensitive to different therapeutic arginine-depleting agents will be discussed in Tables 2 and 3. Scientists have been developing arginine depleting agents in an attempt to deprive cancer cells of arginine. Since decades ago, there have been reports showing arginine depletion by different forms of arginase-induced cell death in different kinds of cancer cells, but there were no arginase formulation developed for clinical use at that time [127, 128]. Until recent two decades, two kinds of arginine depleting agents have been developed and under clinical trials. They are pegylated arginine deiminase and pegylated arginase I [13, 129].

**Table 2:** Current progress of pre-clinical studies of ADI-PEG20 in treating cancer

Cancer type tested	Progress	References
Hepatocellular carcinoma (HCC)	1. Decreased HCC cell viability in vitro	[13]
	2. Suppressed tumor growth and prolonged survival of engrafted.c. implanted tumor-bearing SCID mice	
	3. ASS1 + ve and OTC + ve HCC cells are resistant to ADI.	
Melanoma	1. Decreased melanoma cell viability in vitro	[13]
	2. Suppressed tumor growth and prolonged survival of s.c. implanted tumor-bearing athymic nude mice	
Small cell lung cancer	1. Induced autophagy and cell death in ASS1-ve cell in vitro (about 50% of samples tested in the study were ASS1-ve)	[164]
	2. Suppressed growth of s.c. implanted tumor in athymic nude mice	
Glioblastoma	1. Induced autophagy and caspase independent cell death in ASS1-ve cell lines and clinical samples (approximately 30% glioblastoma samples tested in the study are ASS1-ve)	[165]
	2. Autophagy inhibitor chlorquine accelerated ADI-PEG20 induced cell death in vitro	
Pancreatic cancer	1. Inhibited growth and induced apoptosis of ASS1-ve pancreatic cancer cell lines in vitro (about 80% pancreatic cancer samples tested in the study were ASS1-ve)	[166, 167]
	2. Suppressed growth of subcutaneously implanted tumor in athymic nude mice.	
	3. ADI-PEG20 + gemcitabine showed enhanced cell death in gemcitabine-resistant ASS1-ve pancreatic cell line compared to ADI-PEG20 or gemcitabine only groups in vitro	
	4. ADI-PEG20 + gemcitabine enhanced growth suppression in s.c. implanted gemcitabine-resistant ASS1-ve tumor in athymic nude mice.	
Acute myeloid leukemia (AML)	1. Induced primary AML apoptosis in vitro	[137]
	2. Reduced AML burden in NOD-SCID mice	
	3. ADI-PEG20 + cytarabine further enhanced AML clearance	
Prostate cancer	1. Induced autophagy, mitochondrial dysfunction, DNA leakage and caspase-independent cell death in a prostate cancer cell line in vitro	[132, 168]
	2. Autophagy inhibitor chloroquine enhanced and accelerated ADI-PEG20 induced prostate cancer cell death in vitro	
	3. ADI-PEG20 + docetaxel showed enhanced tumor suppression in s.c. implanted tumor in athymic nude mice	
Bladder cancer	1. Induced caspase-independent apoptosis and autophagy in bladder cancer cell lines in vitro and reduced tumor growth and in vivo (s.c. implanted tumor in athymic nude mice)	[169, 170]
	2. ASS1-ve due to methylation may be related poor prognosis clinically, and linked to invasion and enhanced invasion and proliferation in bladder cancer cells in vitro	
	3. Inhibited pyrimidine metabolism by reducing protein level of thymidylate synthase, dihydro-folate reductase and thymidine kinase 1 and enhanced cytotoxicity in ASS1-methylated bladder cancer cell lines in vitro and in vivo (s.c. implanted tumor in CD1 nude mice)	
Breast cancer	Induced mitochondrial damage and autophagy-dependent cell death in ASS1-ve breast cancer cell in vitro	[171]

**Table 3:** Current progress of pre-clinical studies of peg-arg I as anti-cancer agent

Cancer type tested	Progress	References
Hepatocellular carcinoma (HCC)	1. Suppressed HCC cell growth and induced apoptosis in vitro	[136, 172].
	2. Suppressed OTC-deficient tumor growth in athymic nude mice	
Acute myeloid leukemia (AML)	1. Induced necrotic cell death in AML cell lines and some AML patient samples in vitro and in vivo (implantation of HL-60 cell line in to NOD/SCID γ mice through tail vein)	[154]
	2. Peg-arg I + cytarabine enhanced cytotoxicity in AML cell lines and AML patient samples in vitro	
Acute lymphoblastic leukemia (ALL)	1. Induced apoptosis in T-lineage ALL (T-ALL) cell lines in vitro	[153, 155, 173]
	2. Peg-arg I + cytarabine therapy induced T- ALL cell apoptosis in vivo (Peg-arg I monotherapy did not prolong the survival of T-ALL bearing in NOD-SCID mice)	
	3. MSCs protected T-lineage ALL cell lines from peg-arg I cytotoxicity via soluble factors in vitro, pre-treating MSCs with vincristine may suppress such stromal protection	
	4. eIF2α phosphorylation sensitized T-ALL cells to peg-arg I cytotoxicity in NOD-SCID mice	
Glioblastoma	1. Induced ASS1-dependent non-apoptotic cell death which may be enhanced by autophagy inhibitor CQ in glioblastoma cell lines in vitro	[174]
Melanoma	1. Induced S and G_2_/M phases cell cycle arrest and apoptosis in melanoma cell line A375 in vitro	[175]
	2. Suppressed s.c. implanted melanoma in athymic nude mice	
Prostate cancer	Induced autophagic cell death in OTC-ve cells in vitro	[176]
Pancreatic cancer	1. Induced apoptosis in pancreatic cancer cell line Panc-1 in vitro	[172]
	2. Suppressed tumor growth in a s.c. implanted pancreatic cancer in athymic mice model	
Mesothelioma	1. Suppressed growth of different cell lines in vitro and in vivo (s.c. implanted mesothelioma in athymic nude mouse model)	[148]
	2.Induced apoptosis and G1 arrest in mesothelioma cells in vivo	
	3. Peg-arg I, cispatin and premetrexed did not show synergistic effect against mesothelioma growth in vivo	
	4. Peg-arg I depleted serum and intratumoral arginine, and was internalized in mesothelioma cells in vivo	

### Arginine depletion by ADI

ADI breaks down arginine intro citrulline, cells without ASS-1 expression may encounter arginine starvation upon arginine deiminase treatment. Arginine deiminase is derived from micro-organisms and human cells do not produce such enzyme [130]. As a foreign protein, arginine deiminase may induce antibody induction and anaphylaxis, causing undesired adverse effect and shortened half-life of the drug [131]. To solve the problem, arginine deiminase was pegylated to reduce immunogenicity and to lengthen half-life from 4 h in the native form to 6 days in the pegylated form [13]. The ADI developed for therapeutic use is ADI-PEG20. In preclinical studies, ADI-PEG20 was reported to be effectively in suppressing cancer growth, inducing apoptosis and autophagy in different cancer types in vitro and in vivo. Pegylated arginine deiminase induce growth inhibition or even cell death by autophagy and apoptosis [132]. There are pre-clinical reports showing combined anti-tumor effect of pegylated arginine deiminase with other drugs like chloroquine, gemcitabine and TORC1/TORC2 inhibitor P529 in sarcoma, pancreatic cancer and glioblastoma respectively [133–135]. On the other hand, ASS and OTC expressions are suggested to be the culprit of ADI-PEG20 resistance in vitro [13, 136, 137]. The pre-clinical findings are summarized in Table 2.

### Clinical progress of ADI-PEG20 in treating cancer

There have been clinical trials of pegylated arginine deiminase in melanoma, hepatocellular carcinoma, mesothelioma, and some other advanced cancers patients [138, 139]. In the phase I/II clinical trials of ADI-PEG20 on melanoma, it was well tolerance without serious adverse reactions [140, 141]. Drop of plasma NO level was displayed in patients treated with ADI-PEG20 [140]. Neutralizing antibody production was detected in some patients’ plasma during drug treatment. However, arginine level depletion in patients sustained for about a week and returned to baseline level, suggesting that resistance on ADI-PEG20 may arise upon prolonged drug treatment [140, 141]. Although not the focus of phase I/II trials, patients treated with ADI-PEG20 did not show any reduction in the melanoma progression and whether the treatment may prolong survival seems to depend on the disease stages during treatment [140, 141]. In phases I/II trials of ADI-PEG20 in advanced hepatocellular carcinoma, significant but transient plasma arginine depletion was observed in patients treated with ADI-PEG20. Neutralizing antibody production was noted along treatment although severe adverse reactions were not observed [26, 131]. This suggests drug resistance may arise upon prolonged treatment due to neutralizing antibody production and such phenomenon may explain the negative correlation between plasma neutralizing antibody level and duration of arginine depletion upon ADI-PEG20 treatment. ADI-PEG20 treatment may lead to stable disease but reduction in tumor progression was not observed, although these phenomena may require larger scale of study with longer observation period for further verification [26, 131]. Duration of overall survival of patients may be positively correlated with the duration of plasma arginine depletion [26]. In a phase II randomized clinical trial of ADI-PEG20 on mesothelioma patients, ADI-PEG20 improved PFS in patients with ASS1-deficient mesothelioma and the adverse effects observed in patients were tolerable [139]. But consistent with other trials, neutralizing antibodies against ADI-PEG20 was observed in some patients in later part of treatment, leading to resistance [139]. In another pilot-scale phase I clinical trial using ADI-PEG20 with docetaxel on different solid tumors like non-small cell lung cancer, castrate resistant prostate cancer and head and neck squamous cell carcinoma [142]. In the study, patients receiving ADI-PEG20 and docetaxel treatment displayed significant drop in plasma arginine level for the first 2–3 months of treatment, rise of arginine level, and ADI-PEG20 antibody at the later stage of treatment and tolerable adverse effect throughout the treatment [142]. These studies suggest using arginine depletion as an anti-cancer treatment may be safe with little adverse effect and may benefit the patients by prolonging overall survival. However, neutralizing antibodies against ADI-PEG20 were observed in patients of many clinical trials. This may limit the therapeutic potential of ADI as an arginine depleting agent for cancer treatment. In a dose escalation study of ADI-EGP20 together with cisplatin and pemetrexed in thoracic cancer patients, sustainable arginine depletion was observed in all patients and neutralizing antibodies level was much lower compared to ADI-PEG20 mono therapy or ADI-PEG20, docetaxel combinational therapy [143]. This discovery suggests that in the future, immune-suppressive agents may be used together with ADI-PEG20 for drug resistance due to ADI-PEG20 neutralizing antibodies upon prolonged treatment.

### ADI-PEG20 resistance and ways to enhance efficacy of ADI-PEG20 as anti-cancer therapy

Other than neutralizing antibody production in patients receiving ADI-PEG20 treatment, there are other intrinsic ADI-PEG20 resistance mechanisms found in cancer cells discovered in pre-clinical studies. Autophagy and enhanced ASS1 expression are known to be associated with ADI-resistance [133, 144]. In an ASS1^low^ sarcoma in vivo model reported, ADI-PEG20 showed significant reduction in tumor growth compared to control, but a slow increase in tumor size was still observed over time and the tumor lysate showed significantly enhanced ASS1 expression [133]. In the same report, ADI-PEG20 treated sarcoma cells in vitro showed marked increase of autophagosome compared to control [133]. The autophagy inhibitor chloroquine (CQ) combined with ADI-PEG20 significantly enhanced the cytotoxicity in sarcoma cells in vitro and CQ together with ADI-PEG20 induced sarcoma volume significantly in vivo [133]. Such finding suggests the supportive role of autophagy in ADI resistance.

Some other conditions like hypoxia and enhanced glutaminolysis may induce ASS1 expression in ASS1-ve cancer cells [145, 146]. A study demonstrated hypoxia-induced ASS1 expression in ADI-PEG20 treated breast cancer cell lines in vitro, which may be associated with ADI resistance [146]. In another study of ADI-resistance using melanoma cell lines, all ADI-resistant cells displayed enhanced ASS1, GLS1, and GDH protein expressions mediated by up-regulated c-myc, comparing to parental cell lines [145]. Those ADI-resistant cell lines were found sensitive to PI3K/Akt, glycolytic inhibitors, and glutaminase inhibitors, suggesting a correlation between Warburg effect and ADI-PEG20 resistance [145]. Such correlation is also reported in another study by metabolite profiling of cancer cells in vitro [147]. In that study, arginine depletion inhibited Warburg effect, reduced aerobic glycolysis, increased glutamine anaplerosis, oxidative phosphorylation, and serine biosynthesis together in different cancer cell types in vitro [147]. ADI-PEG20 and glutamine metabolism inhibition by BPTES and silencing GLS expression induced synthetic lethality and significantly reduced tumor growth in melanoma xenograft in vivo [147]. These findings suggest an interaction between glutamine and arginine metabolism in cancer cells. Arginine depletion and glutamine metabolism inhibition may work synergistically in cancer treatment.

In another recent study on mesothelioma, a weak point of ASS1-deficiency was reported [148]. In the study, ADI-PEG20 treated ASS1-deficient mesothelioma cells showed decreased polyamine metabolite and enhanced polyamine synthetics enzymes in vitro [149]. Similar finding was observed clinically. A decreased level of polyamine was observed in mesothelioma patient’s plasma after ADI-PEG20 treatment [149]. Furthermore, when ASS1- deficient mesothelioma cells were treated with ADI-PEG20 and ODC inhibitor DFMO together, synthetic lethality was observed in vitro [149]. The research findings suggest arginine degrading enzymes may have synergistic effects with many other pathway inhibitors in treating arginine-auxotrophic cancers.

Interestingly, there may be conditions that ASS1 does not re-express even upon arginine depletion by ADI-PEG20 [150]. In a BRAFi resistant melanoma cell model, c-myc proteins were actively degraded, that led to in-ability to re-express ASS1 [150]. Autophagy was also ineffective in BRAF inhibitor resistant melanoma cells due to attenuated level of autophagy associated proteins [150]. Therefore, BRAFi-resistant melanoma cells expressed very low level of ASS1 and were very sensitive to ADI-PEG20 in vivo [150]. This finding provides a clue of using ADI-PEG20 in treating particular cancer types which have impaired c-myc.

### Arginine depletion by pegylated arginase I

Another arginine depleting agent under clinical trial is pegylated arginase I. Pegylated arginase I breaks down arginine to ornithine. Pegylated arginine deiminase targets cancer cells defective in ASS1 expression, while pegylated arginase I targets a broader spectrum of cancer cells, which are defective in OTC and/or ASS1 expression. Pegylated arginase I has advantage over the native human arginase because pegylated arginase I have extended half-life from a few hours to 3–4 days [136]. There are two forms of pegylated arginase I developed by different parties. One form involves pegylation of human native arginase I [129]. The other form involves replacement of manganese (II) ion prosthetic group of native human arginase I by cobalt (II) ion and pegylation of the modified enzyme [151]. The prosthetic-group modified pegylated arginase I is said to have further enhancement of enzyme stability, while there is claim that early pre-clinical studies of such modified formulation may be significantly more toxic than the pegylated native arginase I [151, 152]. Nonetheless, both forms of pegylated arginase I are reported to be effective anti-cancer agent by suppressing cancer growth and causing apoptosis in vitro and in vivo. In our previous study, pegylated arginase I treatment may reduce OTC protein expression in human T-ALL cell lines and also hMSCs [153]. There are also reports that pegylated arginase I were internalized in AML blasts in vitro and mesothelioma in vivo and could deplete intra-cellular and intratumoral arginine respectively [148, 154]. These findings suggest the high potency of pegylated arginase I in arginine depletion. The pre-clinical findings are summarized in Table 3.

### Ways to enhance efficacy of pegylated arginase I as anti-cancer therapy

Just like pegylated arginine deiminase, pegylated arginase I may induce growth suppression and probably autophagy and/or apoptosis, depending on cancer types. There are pre-clinical data showing pegylated arginase I may show stronger anti-cancer effect in combination with other chemotherapeutic agents. Pegylated arginase I and 5-flurouracil showed more potent tumor suppression than pegylated arginase I alone in a subcutaneous hepatocellular carcinoma mouse model [136]. Pegylated arginase I in combination with cytarbine showed enhanced cytotoxicity in T-lineage ALL (both in vitro and in vivo) and more recently in AML (both in vitro and in vivo) [154, 155]. However, near total clearance of cancer cells using pegylated arginase I has not been demonstrated in any of the studies listed above. This implies some cancer cells are resistant to pegylated arginase I. Currently, there is no well-established mechanisms of pegylated arginase I resistance. A recent study identified some possible candidate resistance genes of pegylated arginase I in cancer patient samples. Interestingly, in the study, expression level of arginine recycling or transport molecules in leukemic blasts did not correlate with sensitivity to pegylated arginase I [154]. Instead, enhanced epidermal growth factor isoform epiregulin was observed in pegylated arginase I-sensitive leukemic blasts and enhanced heat shock protein HSPA6 was observed in pegylated argianse I-resistant leukemic blasts [154]. Such findings may provide an insight to the design of drug combination protocols. Apart from intrinsic resistance, cancer micro-environment may also contribute to pegylated arginase I resistance. Our in vitro study suggested that mesenchymal stromal cells may protect T-ALL blasts from cytotoxicity induced by pegylated arginase I. However, suppressing mesenchymal stromal cells with vincristine may reverse the cytotoxicity of pegylated arginase I to T-ALL blasts [153].

### Clinical progress of pegylated arginase I in treating cancer

There is one clinical trial of pegylated arginase I on hepatocellular carcinoma. The form of pegylated arginase I used is the pegylated formulation without modification of prosthetic group. In the 8 week study of advanced hepatocellular carcinoma patients, patients taken pegylated arginase I displayed mainly grade 1 and grade 2 toxicities without severe adverse effects I. These adverse effects disappear after discontinuing pegylated arginase I. Unlike patients treated with pegylated arginine deiminase, no neutralizing antibodies were detected in sera of patients receiving pegylated arginase I. Throughout the 8 weeks of study, arginine depletion was found sustainable in all patients treated with pegylated arginase I, apparently the patients did not develop resistance to pegylated arginase I. However, due to the insufficient patient samples as well as the relatively advanced stage of the disease being treated, there is no definite conclusion on the treatment response of the patients to pegylated arginase I [27]. The low adverse effects observed in patients treated with pegylated arginase I suggest pegylated arginase I may be a good candidate as an arginine depletor for cancer treatment. Comparing to ADI-PEG20, there are less publications on the anti-cancer mechanisms of pegylated arginase I, but some of the research findings on arginine depletion using ADI-PEG 20 may also be applicable to pegylated arginase I. Further pre-clinical studies and clinical trials are required to obtain more information on the science, efficacy and optimal dosage of this approach.

### Other arginine depletors

Apart from ADI-PEG20 and pegylated arginase I, there are some other formulations recently developed for arginine depletion therapy. They are the ADI-cell penetrating fusion protein complex, engineered arginase-fc protein and arginine decarboxylase. Unlikely pegylated arginase I, ADI-PEG20 is poorly internalized in cells and it mainly allows extra-cellular arginine depletion, therefore ASS1-expressing cells may be resistant to ADI-PEG20 [146, 154]. ADI-cell-penetrating fusion protein complex is designed to overcome ADI-resistance in ASS1-expressing cancer cells. ADI-cell penetrating fusion protein complex is a recombinant fusion of recombinant ADI and a pH-sensitive cell penetrating protein complex [146]. This formulation allows both extracellular and intracellular depletion arginine depletion and was reported to overcome ADI-resistance and induce cytotoxicity in ASS1-expressing breast cancer in vitro [146]. Arginase FC fusion protein is a genetically engineered protein produced by a human arginase and a Fc chain fusion gene construct [156]. Fusing the arginase to Fc region of an immunoglobulin G may prolong the half-life of the fusion protein [156]. Arginase-fc demonstrates arginine depletion activity equivalent to native arginase. Arginase-fc also demonstrates anti-tumor activity in hepatocellular carcinoma in vitro and in a subcutaneous mouse xenograft model [156]. Another formulation of arginine depletor is the arginine decarboxylase (ADC). ADC had not been identified in any mammalian cells, until the human ADC gene was cloned some years ago [157]. The formulation mentioned as anti-cancer drug candidate was originated and isolated from *E. coli* [157–159]. Arginine decarboxylase breaks down arginine into agmatine, which may be converted into polyamines. The purified arginine decarboxylase demonstrates anti-cancer activity in Hela cells, L1210 (mouse lymphoblastic leukemia) and colorectal cancer cell line by inducing cell cycle arrest and apoptosis in vitro [158, 159]. The short half-life of ADC in vivo is now a major obstacle in using it as an anti-cancer drug [159]. Chemical modification may help to solve this problem. Nonetheless, ADI-cell penetrating fusion protein complex, arginase-fc protein and ADC require further investigation for the efficacies in arginine depletion cancer therapy.

In summary, arginine depletion may be a feasible approach for cancer treatment because of the anti-cancer effects reported in pre-clinical studies, the low adverse reactions and the treatment-achieved stable disease in clinical trials of advanced stage cancers. However, the choice of arginine depleting agent for such use may require further clinical investigation. A therapeutically useful arginine depleting agent for cancer treatment should have low toxicity, non-immunogenic (to prevent antibody production and allergic reactions) and fast-acting (to delay emergence of drug resistance) with long circulation half-life (to achieve sustained arginine depletion). Furthermore, there is no study showing complete remission of tumor by using arginine depletion alone. This suggests the possibility of emergence of resistance to arginine depletion, probably by re-expression of ASS1 or autophagy. For the better benefit of patients, it is important to investigate the resistance mechanisms of arginine depletion and also the potential synergy of arginine depletion with other drugs to maximize therapeutic efficacy and minimize the therapy related toxicity.

## Conclusions

With different approaches in interrupting amino acid metabolism in cancer cells, enzymatic depletion strategy is the most well-studied and promising. The hydrolytic enzymes can extensively eliminate the target amino acids to ensure specific amino acid depletion in blood circulation. With the previous research outcome mentioned above, we come up with several conclusions. Firstly, among all the glutamine metabolism inhibition treatments, glutaminase inhibition is apparently feasible due to the high specificity, efficacy and well tolerance shown in pre-clinical studies and clinical trials. The side effects of glutamine depletion using hydrolytic enzymes are too severe, and there isn’t glutamine-specific hydrolytic enzyme yet, making it currently not a good approach for treating cancer patients. Glutamine transporter inhibitors currently developed require too high dosage and development of transporter inhibitors with higher potency may be required. Secondly, asparaginase depletion using L-asparaginase has been a very effective approach in treating hematologic malignancies with detailed toxicity profile and well-studied resistance mechanism. L-asparaginase demonstrated anti-cancer function even to solid tumors. However, the usage of L-asparaginase is currently limited to hematologic malignancies as the side effects may out-weight the benefits especially in adult patients. These side effects are mainly due to the anaphylaxis induction and off-target glutamine depletion in clinical use. Thirdly, although pegylated arginine deiminase and pegylated arginase I are both arginine depletors, arginine deiminase is foreign to human body and may not achieve sustainable arginine depletion in blood circulation. Further modification on ADI-PEG20 or proper drug combination may help overcoming the antibody-induced resistance to ADI-PEG20 and allow sustainable plasma arginine depletion. Pegylated human arginase I, though lacking clinical data on efficacy over a broad range of cancer types, it shows good safety profile and sustainable arginine depletion in patients. This suggests pegylated arginase I will be a suitable candidate for anti-cancer therapy, but more research is required to explore the potential use of pegylated arginase I in treating different cancer types.

In the future design of amino acid starvation treatment against cancer, there are several important aspects to consider. Firstly, the auxotrophy of cancer cells to particular amino acid(s) should be thoroughly investigated for the choice of amino acid target. The most ideal target will be an amino acid auxotrophic to cancer cells but not normal cells, and at the same time the amino acid is very important for cancer growth. Next, the enzyme is preferably of human origin. This may reduce the anaphylactic response and also antibody reaction. The use of human originated enzymes may help to reduce the side-effects and also drug resistance due to autoantibodies. Furthermore, the enzyme for amino acid depletion should be pegylated to prolong the half-life and reduce the immunogenicity. This may increase the durability of the enzyme in patients’ circulation to allow sustainable amino acid depletion. The side-effects due to anaphylaxis may also be reduced. Last but not least, amino acid depletion should combine with other chemotherapeutic agents to enhance efficacy. Multi-drug protocols should be designed to provide effective and safe treatment regimen based on their complementary mechanisms.

## Abbreviations

ADC: Arginine decarboxylase
ADI: Arginine deiminase
ADI-PEG20: Pegylated arginine deiminase
ALL: Acute lymphoblastic leukemia
AML: Acute myeloid leukemia
AOC: Aminooxetane-3-carboxylate
ASL: Arginine-succinate lyase
ASNS: Asparagine synthetase
ASS1: Arginino-succinate synthetase
ATP: Adenosine triphosphate
BPTES: Bis-2-(5-phenylacetamido-1,2,4-thiadiazol-2-yl)ethyl sulfide
BPTES-NP: BTPES nanoparticle
B-Raf: v-raf murine sarcoma viral oncogene homolog B1
BRAFi: B-Raf inhibitor
CQ: Chloroquine
DFMO: D,L-α-difluoromethylornithine
DNA: Deoxyribonucleic acid
DON: 6-Diazo-5-oxo-L-norleucine
*E. coli*: *Escherichia coli*
eIF2α: Eukaryotic initiation factor 2 alpha
GDH: Glutamate dehydrogenase
GLS: Glutaminase
GLS1: Kidney-type glutaminase
HCC: Hepatocellular carcinoma
Hsp60: Heat shock protein 60
IDH: Isocitrate dehydrogenase
MSC: Mesenchymal stromal cells
mTORC1: Mammalian target of rapamycin complex 1
NO: Nitric oxide
NOD-SCID mouse: Non-obsese diabetes severe combined immune-deficiency mouse
NOS: Nitric oxide synthases
NOS: Nitroc oxide synthase
ODC: Ornithine decarboxylase
OTC: Ornithine transcarbamylase
pegylated arginase I: Peg-arg I
PI3K-Akt Pathway: Phosphatidylinositol 3-kinase-protein kinase B pathway
s.c.: Sub-cutaneous injection
SLC1A5/38A2: Solute carrier family 1 member 5/38A2
T- ALL: T-lineage acute lymphoblastic leukemia
TCA cycle: Tricarboxylic acid cycle
TORC1: Target of rapamycin complex 1
TORC2: Target of rapamycin complex 2
**γ**-FBP: (R)-gamma-(2-fluoro-benzyl)-L-proline
